# Tear fluid proteomics multimarkers for diabetic retinopathy screening

**DOI:** 10.1186/1471-2415-13-40

**Published:** 2013-08-07

**Authors:** Zsolt Torok, Tunde Peto, Eva Csosz, Edit Tukacs, Agnes Molnar, Zsuzsanna Maros-Szabo, Andras Berta, Jozsef Tozser, Andras Hajdu, Valeria Nagy, Balint Domokos, Adrienne Csutak

**Affiliations:** 1 Department of Computer Graphics and Image Processing, Bioinformatics Research Group, University of Debrecen, Faculty of Informatics, Debrecen, Hungary; 2 NIHR Biomedical Research Centre for Ophthalmology, Moorfields Eye Hospital NHS Foundation Trust and UCL Institute of Ophthalmology, London, UK; 3 Department of Biochemistry and Molecular Biology, Proteomics Core Facility, University of Debrecen, Medical and Health Science Center, Debrecen, Hungary; 4 Centre for Research on Inner City Health, Keenan Research Centre Li Ka Shing Knowledge Institute, St Michael’s Hospital, Toronto, Ontario, Canada; 5 Department of Ophthalmology, University of Debrecen, Medical and Health Science Center, Debrecen, Hungary; 6 Astridbio Ltd, Debrecen, Hungary; 7 InnoTears Ltd, Debrecen, Hungary

**Keywords:** Diabetic retinopathy screening, Tear fluid biomarkers, Quantitative mass spectrometry, Pattern recognition

## Abstract

**Background:**

The aim of the project was to develop a novel method for diabetic retinopathy screening based on the examination of tear fluid biomarker changes. In order to evaluate the usability of protein biomarkers for pre-screening purposes several different approaches were used, including machine learning algorithms.

**Methods:**

All persons involved in the study had diabetes. Diabetic retinopathy (DR) was diagnosed by capturing 7-field fundus images, evaluated by two independent ophthalmologists. 165 eyes were examined (from 119 patients), 55 were diagnosed healthy and 110 images showed signs of DR. Tear samples were taken from all eyes and state-of-the-art nano-HPLC coupled ESI-MS/MS mass spectrometry protein identification was performed on all samples. Applicability of protein biomarkers was evaluated by six different optimally parameterized machine learning algorithms: Support Vector Machine, Recursive Partitioning, Random Forest, Naive Bayes, Logistic Regression, K-Nearest Neighbor.

**Results:**

Out of the six investigated machine learning algorithms the result of Recursive Partitioning proved to be the most accurate. The performance of the system realizing the above algorithm reached 74% sensitivity and 48% specificity.

**Conclusions:**

Protein biomarkers selected and classified with machine learning algorithms alone are at present not recommended for screening purposes because of low specificity and sensitivity values. This tool can be potentially used to improve the results of image processing methods as a complementary tool in automatic or semiautomatic systems.

## Background

### Diabetic retinopathy screening

Diabetic retinopathy (DR) is the most common complication of diabetes mellitus and is currently the leading cause of blindness in the economically active population in developed countries [1]. The initially latent disease could lead to vision loss without any symptoms initially. Timely diagnosis and therapy however can significantly decelerate its progress, necessitating regular DR screening or appropriate follow-up in all patients with diabetes. Screening can be carried out by direct and indirect ophthalmology or increasingly by using photographic methods [2].

The effectiveness of different screening modalities has been widely investigated. UK studies show sensitivity levels for the detection of sight-threatening diabetic retinopathy of 41-67% for general practitioners, 48-82% for optometrists, 65% for ophthalmologists, and 27-67% for diabetologists and hospital physicians using direct ophthalmoscopy [3,4]. Sensitivity for the detection of referable retinopathy by optometrists have been found to be 77-100%, with specificity of 94-100% [5].

Photographic methods currently use digital images with subsequent grading by trained individuals. Sensitivity for the detection of sight-threatening diabetic retinopathy have been found 87-100% for a variety of trained personnel reading mydriatic 45° retinal photographs, with specificities of 83-96% [6]. The British Diabetic Association (Diabetes UK) has established standard values for any diabetic retinopathy screening programme of at least 80% sensitivity and 95% specificity [7,8].

Regular DR screening is centralized in several developed countries due to cost-efficiency and quality control issues [9]. Digital fundus images are captured at the place of patient care and forwarded to a grading center for evaluation by specially trained human graders or ophthalmologists [10]. The system operates with high accuracy but due to its labor intensive nature, it might be poorly scalable in economically challenged countries [11].

In order to improve scalability and cost-effectiveness, many research groups are working on developing automated image analysis technologies [12]. Introducing these technologies in DR screening could substitute first phase examinations performed by human graders. Following automated pre-screening, human graders would only have to examine images that are either questionable or true positive, and potentially carry out quality control on a subset of those deemed normal by the software [13]. Preliminary results are promising, sensitivity and specificity indicators of automated systems are close to that of human graders [14-16]. An international Retinopathy Online Challenge is available in order to compare the results of image processing based algorithms for DR identification via mycroaneurism detection. The system developed by our team currently performs the best on this challenge [17].

The aim of this paper is to describe a pilot study, conducted as a first attempt to examine the use of tear fluid proteomics for DR pre-screening. Our hypothesis was that it is possible to categorize patients with unknown clinical status into a DR or a non-DR group, based on the protein pattern identified in the tear fluid sample.

### Tear fluid proteomics

Besides computer based image processing other methods can also support DR screening. Tear fluid proteomics is such a possible novel tool for population screening, which is based on the pre-screening of a large number of patients and uses human graders only in positive or ambiguous cases. The protein composition of tear fluid has been investigated by numerous research groups and more than 500 proteins are already proven to be present there [18,19]. Reports confirm protein profile changes in tear fluid under irregular physiological and pathological conditions (like wound healing or inflammatory diseases etc.) [20]. Besides the proteins secreted by the lacrimal glands, tear fluid might contain proteins from epithelial cells covering the eye surface. Furthermore proteins normally residing in the blood can get into the tear fluid through increased permeability of the conjunctival vessels [21].

Physiological and pathological conditions of the retina also induce changes in the protein composition of the vitreous humour, especially when the blood-retinal barrier (BRB) is damaged [22]. Breakdown of the inner endothelial BRB, occurs in diabetic retinopathy, age-related macular degeneration, retinal vein occlusions, uveitis and other chronic retinal diseases [23]. DR is a complex disease, characterized by vascular alterations, inflammation, and neuronal cell death that involve heat-shock and crystalline proteins. The central mechanism of altered BRB function is a change in the permeability characteristics of retinal endothelial cells caused by elevated levels of growth factors, cytokines, advanced glycation end products, inflammation, hyperglycemia and loss of pericytes. Subsequently, paracellular but also transcellular transport across the retinal vascular wall increases via opening of endothelial intercellular junctions and qualitative and quantitative changes in endothelial caveolar transcellular transport, respectively. Functional changes in pericytes and astrocytes, as well as structural changes in the composition of the endothelial glycocalyx and the basal lamina around BRB endothelium further facilitate BRB leakage. As Starling's rules apply, active transcellular transport of plasma proteins by the BRB endothelial cells causing increased interstitial osmotic pressure is probably the main factor in the formation of macular edema [23]. Recent studies using whole proteome analysis demonstrate that general stress response lead to the induction of heat-shock proteins. The alpha-crystallin is expressed in the retina and over-expressed during diabetes as an adaptive response of retinal cells [24]. These changes are more specific to DR than alteration of tear fluid proteome. However, the invasive sampling method makes it difficult to introduce vitreous humour proteomics in the daily clinical practice.

According to recent publications the protein composition of tear fluid reflects normal or abnormal conditions, which justifies the use of tear fluid for screening purposes. Electrophoresis and chromatography also support that protein patterns of healthy and diabetic people are significantly different [25,26].

Qualitative and quantitative methods used for tear fluid examinations include one and two dimensional electrophoresis, enzyme-linked immunosorbent assay and high throughput liquid chromatography [27-29]. Recently, high sensitivity and high resolution procedures were used for the assessment of the effects of trauma and various diseases in tear fluid of microliter quantity. In these cases, protein profiles were characterized by mass spectrometers MALDI-TOF, SELDI-TOF and LC/MS [30-33].

In a previous study we examined the changes of tear protein concentrations in DR aiming to identify and characterize proteins–potential biomarkers-present in tear fluid in DR patients. Tear fluid samples were examined by quantitative mass spectrometry, and 6 potential biomarkers namely lipocalin 1, lactotransferrin, lacritin, lysozyme C, lipophilin A and immunoglobulin lambda chain were identified. The results of that study have already been published elsewhere [34].

In this paper, we evaluate the usefulness of the previously identified DR marker proteins in tear fluid sample for diagnostic purposes and introduce a method based on whole protein pattern analysis, which can be integrated into the screening of DR as a pre-screening procedure.

### Machine learning

Machine learning is an area of artificial intelligence. IT systems using machine learning methods are capable to learn from training datasets and predict possible outcomes based on new observations. In our case the input data are coming from proteomics experiments and the predicted outcomes are diseased and non-diseased cases (DR or Non-DR). In the learning phase both input data and outcomes are used by the system. During the pre-screening process the trained machine learning system will classify cases into diseased or non-diseased groups.

In our pilot study, we intended to ensure the objectivity of the assessment by using the following 6 different machine learning methods: Support Vector Machine (SVM), Recursive Partitioning (rpart), Random Forest (randomForest), Naive Bayes (naïveBayes), Logistic Regression (logReg), K-Nearest Neighbor (k-NN). Possible positive and negative cases for DR can be identified by machine learning algorithms, allowing the pre-screened population (possible positives) to undergo human screening by using digital retinal imaging. The approach presented in this paper is unique in nature, as no previous study was found in the scientific literature investigating the applicability of tear fluid proteomics based methods for DR screening.

## Methods

### Patient examination

119 patients with diabetes were enrolled in the study. In case of 73 patients one of the eyes were examined due to difficulties in tear fluid sampling (e.g. keratoconjunctivitis sicca, operated eye, noncompliance) or because the retinal image couldn’t be taken (e.g. cataract, hemorrhage, angle-closure glaucoma) or assessed (technical difficulties). Diabetic retinopathy was diagnosed by capturing 7-field fundus images of the patients, evaluated by two independent ophthalmologists. Megaplus Camera Model 1,6i/10 BIT Zeiss (Carl Zeiss Ophthalmic System A6, Jena, Germany) was used for taking images. Out of the 165 eyes examined, 55 were diagnosed healthy and 110 showed signs of DR. One patient had DR in one eye only.

Tear fluid samples were collected from the examined eyes by a trained assistant under standardized conditions [35]. Tear samples were collected with glass capillaries immediately before the pupil dilatation for fundus examination under slit lamp illumination from the lower tear meniscus (a horizontal thickening of the pre-corneal tear film by the lower margin) at the lateral canthus. Care was taken not to touch the conjunctiva. The duration of the sampling process was recorded and the secretion rate was calculated in microl/min, dividing the obtained tear volume by the time of sample collection. Samples used in this investigation had secretion rates of 5–15 μl/min. Samples were centrifuged (1800 rpm) for 8–10 minutes immediately after sample collection, supernatants were deep-frozen at −80°C and were thawed only once for measurements. Tear samples were examined using state-of-the-art nano-HPLC coupled ESI-MS/MS mass spectrometry protein identification as described elsewhere [29]. During the protein-based screening procedure pre-proliferative and proliferative retinopathy patients were both considered important, the term ’patient’ includes both groups diagnosed by using retina images. Global pattern of protein concentrations and its changes were described by the examination of the concentrations of 34 different proteins.

### Application of machine learning methods

By using machine learning algorithms we intended to predict the possible fulfillment of certain future events–using empirical data containing incomplete information. In the present special case, input data (features) are protein levels measured in tear fluids from patients with diabetes and clinical data regarding their DR status (dichotomous variable of none or some). As the method is designed to be used in screening in the future, high sensitivity values were considered as the primary criteria for the construction of the classifier. This was aimed at minimizing the number of false negative cases, thus reducing the chance for missing sight threatening disease. The goal of this machine learning approach is to assess the best classification performance achievable by fitting different models to the dataset (model selection). In order to reach the best possible results, we tested several classifying algorithms (SVM, rpart, randomForest, naiveBayes, logReg, k-NN).

A further objective was to design a marker score for marker selection, targeting the best performance of the tested models. In order to guarantee comparability at the parameter adjustment of the different classifiers, we applied the settings providing the best performance regarding the particular model. In order to monitor the classifier’s performance on the dataset, the testing was accomplished with *k*-fold cross validation procedure [36]. During the *k*-fold cross validation the data set is divided into *k* equal parts. The first *k*-1 set (training set) is used for model construction and later on it is tested on the *k*-fold set (test set). In the further *k*-1 iteration the same procedure is followed, on the first *k*-2 and *k*-fold set the model is learning and on the *k*-1 it is validated. At the end of the cross validation, the estimate is determined as the mean of the features of the *k*-fold model. In this study 5-fold cross validation was used. Standard measures were applied to assess the performance of the different models, e.g.: specificity, sensitivity, accuracy, F-measure (harmonic mean of precision and sensitivity–as a single measure for the performance of the model).

### Data analysis software tools

We collected experimental (proteomics) and clinical data (DR/Non-DR) in an Excel file which was used as input data for the analysis.

During the data analysis process the R statistical framework and the following packages have been used: Support Vector Machine: “e1071”; Recursive Partitioning: “rpart”; Random Forest: “randomForest”; Naive Bayes: “naiveBayes”; Logistic Regression: “glmnet” and for visualization purpose: “ggplot2” [37]. We have developed our own solution for the K-Nearest Neighbor model.

We ran cross validation using the three different approaches below in order to find the best combination of input data and the different models.

First, we used data from all 34 identified proteins for model development.

However, we also wanted to analyze the changes in the performance of the classifiers if we only use a subset of the data. Thus, second, only 6 marker proteins (out of the 34)–previously extracted by classical statistical methods–were used for decision making, applying the six machine learning algorithms. Third, we wanted to further evaluate the performance of the models by reducing the number of input variables therefore we applied principal component analysis (PCA). In this case, we performed dimension reduction to compress the information included in the original dataset with principal component analysis and used it for the visualization of the complex dataset in a 2D plane. With this method the number of input features could be decreased while as much of the variation in data as possible could be retained. Thus, we had fewer input features which are the linear combinations of the original variables and uncorrelated with each other.

#### Statement of ethics

We certify that all applicable institutional and governmental regulations concerning the ethical use of human volunteers were followed during this research (Regional and Institutional Ethics Committee, Medical and Health Science Center, University of Debrecen).

## Results and discussion

### Evaluation of the six classifiers on the data

Out of the six different machine learning algorithms, rpart methodology was found to be the most efficient if we used the dataset of the six marker proteins identified by classical statistical methods. Standard measures (Table 1) show that the model based on the Recursive Partitioning classifier outperforms the other five models (74% sensitivity, 48% specificity and 65% accuracy; rpart/marker). Naive Bayes method shows higher sensitivity but low specificity (38%), if we were using the marker proteins only as input variables (80%; naiveBayes/marker). Random Forest method performs slightly worse than Recursive Partitioning.

**Table 1:** Performance measures of the six different classifiers

**Model**	**Dataset**	**SENS**	**SPC**	**ACC**	**PREC**	**NPV**	**F1**	**LRP**	**LRN**
**naiveBayes**	orig	0.6991	0.4186	0.6218	0.7596	0.3462	0.7281	1.2025	0.7188
	marker	0.8000	0.3874	0.5064	0.3462	0.8269	0.4832	1.3059	0.5163
	pca	0.6731	0.3365	0.4487	0.3365	0.6731	0.4487	1.0145	0.9714
**kNN**	orig	0.6711	0.5000	0.6667	0.9808	0.0385	0.7969	1.3421	0.6579
	marker	0.6688	0.5000	0.6667	0.9904	0.0192	0.7984	1.3377	0.6623
	pca	0.6643	0.3077	0.6346	0.9135	0.0769	0.7692	0.9596	1.0909
**logReg**	orig	0.6923	0.3846	0.5897	0.6923	0.3846	0.6923	1.1250	0.8000
	marker	0.6615	0.3077	0.6026	0.8269	0.1538	0.7350	0.9556	1.1000
	pca	0.6623	0.0000	0.6538	0.9808	0.0000	0.7907	0.6623	Inf
**randomForest**	orig	0.6929	0.4483	0.6474	0.8462	0.2500	0.7619	1.2559	0.6850
	marker	0.6923	0.4103	0.6218	0.7788	0.3077	0.7330	1.1739	0.7500
	pca	0.6748	0.3636	0.6090	0.7981	0.2308	0.7313	1.0604	0.8943
**rpart**	orig	0.7083	0.4722	0.6538	0.8173	0.3269	0.7589	1.3421	0.6176
	marker	0.7404	0.4808	0.6538	0.7404	0.4808	0.7404	1.4259	0.5400
	pca	0.6935	0.4375	0.6410	0.8269	0.2692	0.7544	1.2330	0.7005
**SVM**	orig	0.6645	0.0000	0.6603	0.9904	0.0000	0.7954	0.6645	Inf
	marker	0.6623	0.0000	0.6538	0.9808	0.0000	0.7907	0.6623	Inf
	pca	0.6623	0.0000	0.6538	0.9808	0.0000	0.7907	0.6623	Inf

Performance measures of the six different classifiers on the different input data: orig-full data set; marker- candidate marker proteins only; pca–PCA transformed data. The meaning of the columns: SENS-sensitivity, SPC-specificity, ACC-accuracy, PREC-precision (positive predictive value), NPV-negative predictive value, F1-F-measure, LRP- likelihood ratio positive, LRN-likelihood ratio negative.

Six proteins were identified as independent biomarkers out of the 34 candidate protein, by using statistical hypothesis testing.

There is additional information in the joint distribution of the whole dataset, thus we wanted to examine the maximum possible performance of the model.

Our reason for examining the performance of the reduced set was to develop a practical method for screening, without compromising the performance of the test.

**Figure 1:**
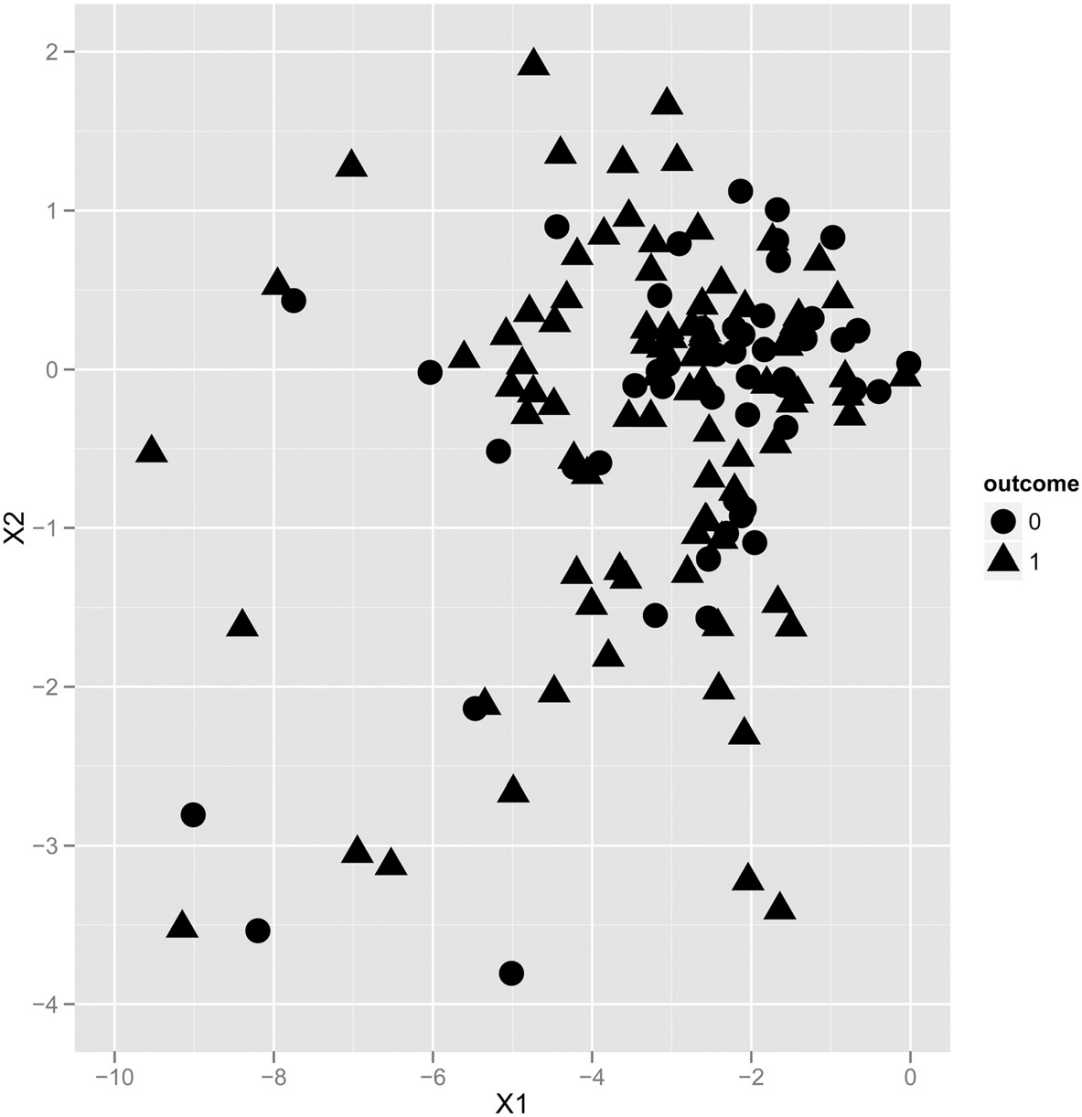
**Scatter plot.** Scatter plot of the PCA transformed data set. X1 and X2 are the two components retained after the transformation. 0 refers to non-DR, 1 refers to DR patients.

We have found that neither the models built on just the 6 marker proteins nor the models built on the PCA preprocessed data performed better or worse than the models built on the whole protein patterns. After Principal Component Analysis the first two components retained 22% of the variation of the original data set. Table 1 shows that in case of retaining the first two components only, performance measures does not change considerably. As a visual presentation of the data we have generated a scatter plot of the first two components. Figure 1 presents the lack of clear decision boundary separating the two classes.

**Figure 2:**
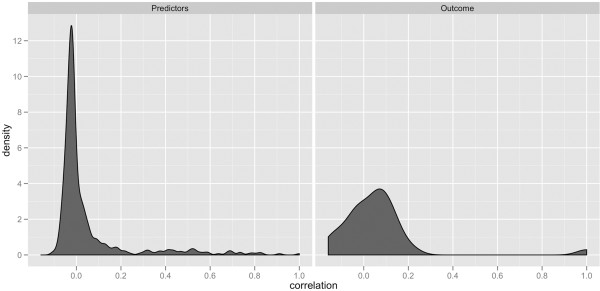
**Probability density function.** Probability density function of the correlation values between predictors (on the left) and between predictors and outcome variables (on the right).

As part of our descriptive analysis we evaluated and visualized the correlation between the predictor variables (protein levels) and also between predictor and outcome variables (DR or non-DR) to have a better understanding on the structure of the data set. According to the probability density function of the correlation values in most of the cases there are low correlation between predictors and predictors/outcomes (Figure 2). These figures suggest that the predictors used separately will not be sufficient for the classification.

### Data set size effect on performance

**Figure 3:**
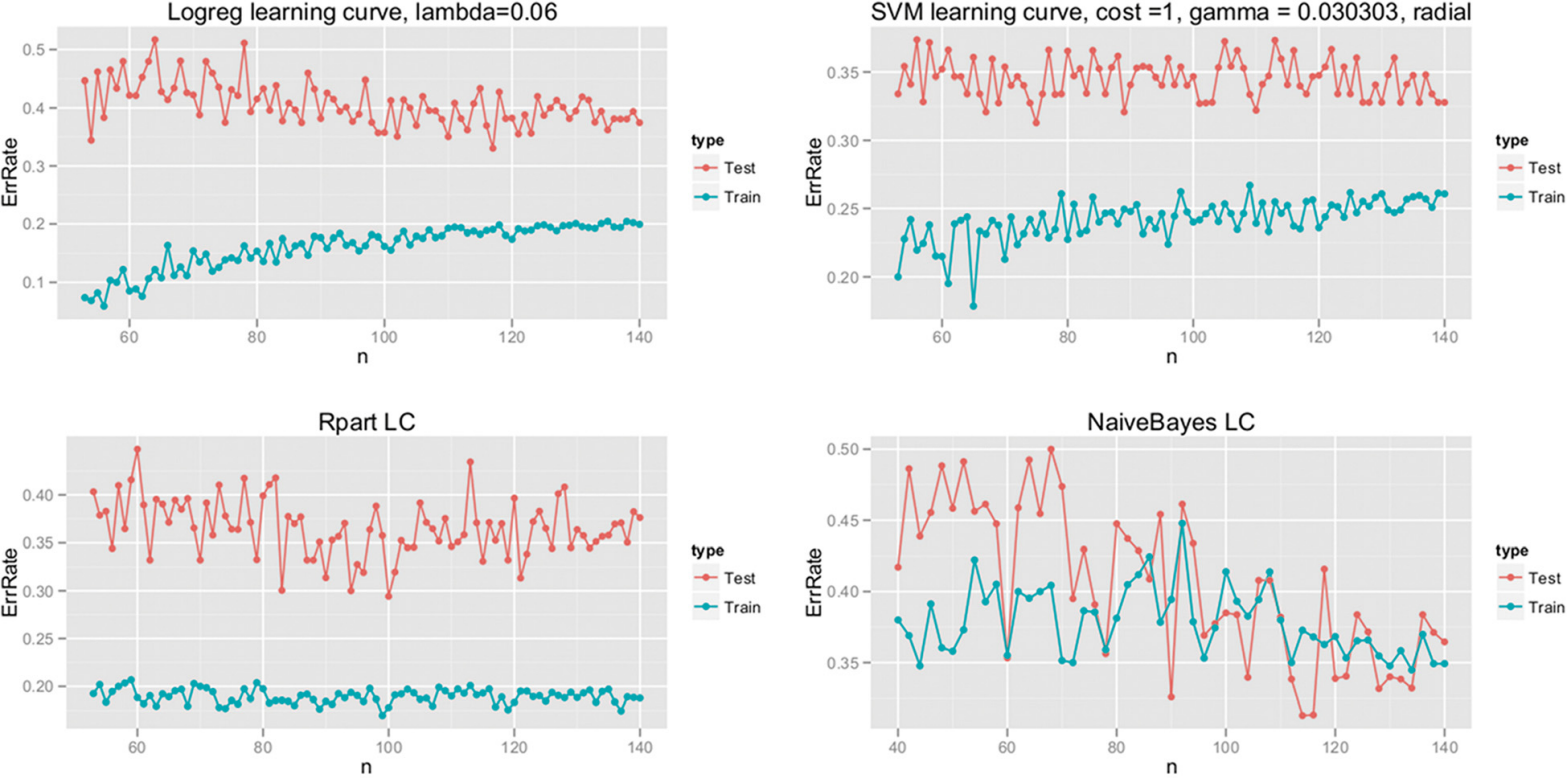
**Learning curves.** Learning curves of Support Vector Machine (SVM), Recursive Partitioning (Rpart) Logistic Regression (Logreg) and Naive Bayes (naiveBayes) classifiers for original data set.

In order to evaluate the impact of the training set size on the reliability of the results we compared the learning curves for each of the cases. With this method we can have an insight to the relation of the bias and variance of the different models and also further improvement possibilities. For more accurate evaluation of the performance of our methods we have to examine if there are any effects of increasing data volume on the performance of the different machine learning methods (Figure 3).

We used learning curve to display the test and training errors for different training set sizes.

The distance of the curves shows a gap between the error rates of training and test set in case of Support Vector Machine (SVM) and Recursive Partitioning (Rpart). This finding suggests that these classifiers have high variance and getting more training data might help with the performance. In case of Logistic Regression (Logreg) and Naive Bayes (naiveBayes) we do not see a gap, but both training and test errors are high, indicating that the models are too simple, thus, we have underfit the data.

## Conclusions

We hypothesized at the beginning of the study, that the system may be utilized for pre-screening purposes, substantially-in long term-reducing the cost of regular screening and the need for health professionals in the process. A possible advantage of this method is that the pre-screening of the risk population (i.e. diabetic patients) can be performed at the patient’s home. Patients can take tear sample by their own, using sampling capillaries delivered by post, and then returning it to the laboratory. Only those with positive screening results should visit a DR screening center.

Based on this pilot study, sensitivity and specificity values of the system examined on a test database were not found high enough to unequivocally support its use as a pre-screening method prior to currently applied screening procedures. However, the results of the system may be further improved by the enlargement of the learning database.

Another promising application of proteomics based machine learning methods is the support of image processing based automated methods by improving their performance to be able to substitute traditional human screening methods in the future. Given constantly dropping price of high throughput technologies, methodologies based on such technologies will be available both for routine point of care diagnostics and screenings [38]. On the other hand, costs of human workforce significantly increase along with the decreasing accessibility of qualified health care professionals. The net result of these forces accelerates the future spread of such technologies.

This study opens up the possibility of including features other than protein analysis: e.g. anamnestic clinical data, results of image processing procedures. Applying more individual parameters as system inputs may considerably improve the sensitivity indicators of a combined system in contrast to the proteomics-alone or image processing-alone systems. This statement is further emphasized as the results of procedures based on image processing are highly promising. The system built on combined procedures may be applied in the future clinical practice.

## Competing interests

The authors declare that they have no competing interests.

## Authors’ contributions

ZST carried out the initiation of the research, management and manuscript writing. TP took part in the patient stratification and manuscript writing. JT and ECS participated in the tear sample processing and proteomics experiments. ET carried out the statistics, the data analysis and the mathematical model development. AMM partook in the evaluation of the validity of screening and manuscript writing. ZSMSZ participated in manuscript writing, clinical data collection and in its integration. AB and VN helped in carrying out the patient examination, the diagnosis, the tear sample collection and photodocumentation. AH performed the mathematical model development. BD carried out the programming, the data analysis and visualisation, the machine learning and mathematical model development. ACS acted as supervisor, took part in project management, helped in patient examination, diagnosis, tear sample collection and photodocumentation. All authors read and approved the final manuscript.

## Authors’ information

Agnes Molnar: CIHR Strategic Training Fellow and Peterborough KM Hunter Charitable Foundation Fellow in the ACHIEVE Research Partnership: Action for Health Equity Interventions.

## Pre-publication history

The pre-publication history for this paper can be accessed here:

http://www.biomedcentral.com/1471-2415/13/40/prepub
